# Skimmianine Modulates Tumor Proliferation and Immune Dynamics in Breast Cancer by Targeting PCNA and TNF-α

**DOI:** 10.3390/ph18050756

**Published:** 2025-05-20

**Authors:** Tuğcan Korak, Hayat Ayaz, Fırat Aşır

**Affiliations:** 1 Department of Medical Biology, Faculty of Medicine, Kocaeli University, 41001 Kocaeli, Turkey; 2 Department of Histology and Embryology, Faculty of Medicine, Dicle University, 21280 Diyarbakır, Turkey; ayazhayat44@gmail.com (H.A.); firatasir@gmail.com (F.A.)

**Keywords:** skimmianine, breast tumor, PCNA, TNF-α, immunohistochemistry, bioinformatics

## Abstract

**Background/Objectives**: Breast cancer continues to be a major global health challenge, driving the urgent need for innovative therapeutic strategies. This study evaluates the anticancer and immunomodulatory potential of skimmianine in breast cancer through a comprehensive approach, integrating biochemical, histopathological, immunohistochemical, and bioinformatics analyses. **Methods**: Thirty-six female Wistar albino rats were divided into three groups: control, 7,12-dimethylbenz[a]anthracene (DMBA)-induced breast cancer, and DMBA + skimmianine (*n* = 12/group). Breast cancer was induced with a single oral dose of 50 mg/kg DMBA in sesame oil. After 16 weeks, skimmianine (40 mg/kg) was administered intraperitoneally for four weeks. Serum CA15-3 levels were measured via enzyme-linked immunosorbent assay (ELISA). Histopathological assessment was performed using hematoxylin and eosin (H&E) staining, and proliferating cell nuclear antigen (PCNA) and tumor necrosis factor-alpha (TNF-α) were evaluated immunohistochemically. Pathway and hub gene analyses were performed using Cytoscape, functional annotation with Enrichr, and immune analyses via the Tumor and Immune System Interaction Database (TISIDB) and Sangerbox. **Results**: The tumor burden in the animals increased after DMBA induction compared to the control groups (0.00 ± 0.00% vs. 89.00 ± 6.60%, respectively, *p* < 0.001), while skimmianine treatment significantly reduced the tumor burden in the animals (49.00 ± 9.40%, vs. DMBA group, *p* = 0.191). Histopathological analysis showed DMBA-induced structural disorganization and malignant clustering, whereas skimmianine preserved ductal structures and mitigated the damage. Compared to the control group, DMBA administration markedly elevated serum CA15-3 levels (0.23 ± 0.06 ng/mL vs. 8.57 ± 1.01 ng/mL, respectively), along with PCNA (13.0 ± 3.0% vs. 25.0 ± 4.0%, respectively) and TNF-α (8.4 ± 1.7% vs. 34.0 ± 5.3%, respectively) expression, indicating active tumor progression. Skimmianine treatment significantly reduced CA15-3 (3.72 ± 0.58 ng/mL), PCNA (20.0 ± 4.1%), and TNF-α (25.0 ± 3.9%) levels (*p* < 0.001). In silico analyses indicated skimmianine’s effects on PCNA influence cell cycle pathways, while TNF-α suppression impacts toll-like receptor (TLR) signaling (adjusted *p* < 0.05). PCNA- and TNF-α-related anticancer effects were especially notable in basal molecular and C2 immune subtypes (*p* < 0.05). Related hub proteins may regulate immune dynamics by reducing immunosuppression and tumor-promoting inflammation (*p* < 0.05). **Conclusions**: Skimmianine shows promise as a breast cancer therapy by simultaneously targeting tumor growth and immune regulation, with PCNA and TNF-α identified as potential key players.

## 1. Introduction

Breast cancer remains a leading malignancy among women worldwide, representing a significant cause of cancer-related mortality. Despite advancements in therapeutic interventions—including surgery, chemotherapy, radiotherapy, hormonal therapy, and targeted treatments—challenges such as therapeutic resistance and adverse side effects persist. Consequently, there is an ongoing necessity to explore novel therapeutic agents that can enhance treatment efficacy while minimizing toxicity [1,2,3,4,5].

Skimmianine (4,7,8-Trimethoxyfuro[2,3-b]quinoline), a naturally occurring furoquinoline alkaloid derived from Dictamnus dasycarpus, has gained significant attention due to its anticancer, anti-inflammatory, and antioxidant properties. Studies have demonstrated its cytotoxic effects across various cancer cell lines, where it induces apoptosis and suppresses cell proliferation [6,7]. Although direct mechanistic investigations in breast cancer remain limited, the quinoline alkaloid class to which skimmianine belongs has been associated with key anticancer activities, including the inhibition of cell migration, suppression of angiogenesis, modulation of nuclear receptor signaling, and induction of cell cycle arrest [8]. Specifically, skimmianine has been reported to inhibit breast cancer cell proliferation while promoting apoptosis through caspase-3 (CASP3)-mediated pathways [9]. Furthermore, its ability to activate nuclear factor erythroid 2-related factor 2 (Nrf2), a critical regulator of detoxification enzymes, suggests a potential role in chemoprevention in breast cancer [10]. Collectively, these findings highlight skimmianine as a promising therapeutic agent targeting key molecular pathways involved in breast cancer progression.

Proliferating cell nuclear antigen (PCNA) is an essential nuclear protein that functions as a processivity factor for DNA polymerase δ, playing a pivotal role in DNA replication and repair. It is an essential proliferative marker that promotes cancer cell division and breast cancer development, while also playing a role in inhibiting apoptosis, thereby contributing to tumor progression [11,12]. An overexpression of PCNA has been observed in various malignancies, including breast cancer, and is often associated with increased tumor aggressiveness and poor prognosis [13]. Tumor necrosis factor-alpha (TNF-α), a key pro-inflammatory cytokine primarily secreted by M1-polarized macrophages, plays a crucial role in maintaining chronic inflammation and contributing to tumor progression [14,15]. One of the key mechanisms of TNF-α in breast cancer involves its ability to promote tumor growth by upregulating the oncogenic protein hepatitis B X-interacting protein (HBXIP), thereby enhancing cell proliferation and survival. TNF-α activates signal transducer and activator of transcription 3 (STAT3), increasing HBXIP promoter activity, while NF-κB and p38 signaling further elevate phosphorylated STAT3 levels. Additionally, HBXIP upregulates TNF receptor 1 (TNFR1), forming a positive feedback loop (TNFR1/NF-κB/p38/p-STAT3/HBXIP/TNFR1) that sustains TNF-α-driven tumor progression in breast cancer [14].

Although previous studies have demonstrated that skimmianine exerts pro-apoptotic effects—primarily through caspase-3 activation—in several cancer cell lines [9], its mechanisms of action in breast cancer have not yet been thoroughly elucidated. To the best of our knowledge, no comprehensive investigation has yet assessed its therapeutic potential in in vivo breast cancer models, particularly regarding its influence on proliferation-associated markers (e.g., PCNA), inflammatory cytokines (e.g., TNF-α), and its immunomodulatory interactions with tumor immune subtypes and cellular infiltration in the tumor microenvironment (TME). To address these gaps, this study aims to investigate the effects of skimmianine on breast cancer in a DMBA-induced rat model by assessing its impact on PCNA and TNF-α expression in tissues, followed by pathway and immunological analyses to explore PCNA- and TNF-α-associated molecular networks using in silico approaches. By integrating histopathological and computational analyses, this study provides the first comprehensive evaluation of skimmianine’s interactions with these key pathways in breast cancer. This will enhance our understanding of skimmianine’s antitumor potential and its regulatory influence on critical molecular mechanisms driving breast cancer progression, ultimately contributing to the development of novel targeted therapeutic strategies.

## 2. Results

### 2.1. Serum Tumor Marker Levels

Serum CA-15 levels are shown in Table 1. DMBA induction caused a significant increase in CA15-3 levels in the rat blood of the DMBA group (0.23 ± 0.06 ng/mL vs. 8.57 ± 1.01 ng/mL, respectively) while skimmianine treatment reduced its levels, suggesting the antitumor activity of the skimmianine treatment (3.75 ± 0.58 ng/mL) (*p* < 0.0001).

### 2.2. Histopathological Findings

**Figure 1:**
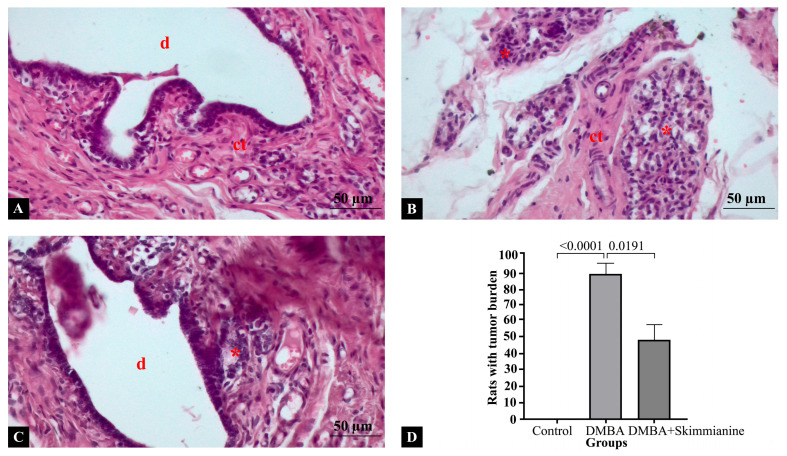
Cross-sections of mammary glands stained by hematoxylin and eosin staining in (**A**) control, (**B**) DMBA, and (**C**) DMBA + skimmianine groups. (**D**) Tumor burden of rats across groups. Ductal structures (d), connective tissue (ct), malignant cells (*). Magnification: 20×.

Histopathological sections of rat mammary glands per group are shown in Figure 1. In the control group, the mammary gland architecture was normal, with well-organized ductal structures with an intact epithelial lining. Connective tissue appeared normal, with no signs of inflammation or abnormal cellular proliferation (Figure 1A). DMBA induction led to a disrupted mammary gland architecture with the presence of dense clusters of malignant cells, indicative of tumor formation. Connective tissue was observed, possibly due to desmoplasia (a fibrotic response to cancer). Increased mitotic activity and nuclear pleomorphism were evident (Figure 1B). Compared to the DMBA group, ductal structures showed improvement with mild alterations. Malignant cell clusters were reduced, suggesting a potential therapeutic effect of the skimmianine treatment. There was less connective tissue disruption compared to the DMBA-induced cancer group and some preservation of mammary gland structure compared to the cancer group (Figure 1C). In the evaluation of the tumor burden across the experimental groups, none of the animals in the control group exhibited histopathological signs of tumor formation, consistent with an intact ductal architecture and the absence of malignant clusters, indicating a 0% incidence of increased tumor burden. In contrast, 89% of the animals in the DMBA-induced group demonstrated a marked increase in tumor burden, characterized by structural disorganization, dense clusters of malignant cells, and elevated PCNA and TNF-α expression levels (*p* < 0.0001). Notably, in the DMBA + skimmianine treatment group, the tumor burden appeared reduced in approximately 49% of the animals, as evidenced by partial preservation of the mammary gland architecture, fewer malignant clusters, and attenuated PCNA and TNF-α immunoreactivity (*p* = 0.191) (Figure 1D). These findings suggest that skimmianine treatment led to a substantial decrease in the proportion of animals exhibiting an increased tumor burden compared to the DMBA group.

**Figure 2:**
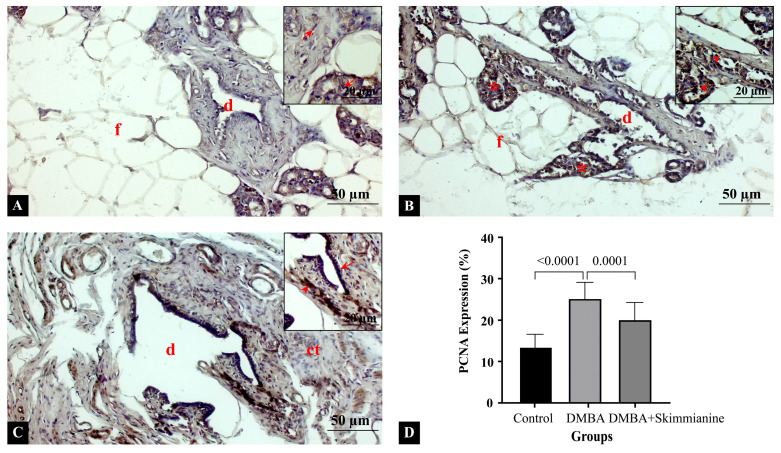
Cross-sections of mammary glands in (**A**) control, (**B**) DMBA, and (**C**) DMBA + skimmianine groups. (**D**) Semi-quantitative measurement of PCNA expression. Ductal structures (d), connective tissue (ct), malignant cells (*), fatty tissue (f), arrow: negative expression, arrowhead: positive expression. Magnification: 20× (low), 40× (insets).

Mammary gland sections were stained with PCNA primary antibody to show cellular proliferation (Figure 2). In the control group, a low PCNA expression showed minimal cell proliferation. Few or no nuclei were stained with PCNA positively, as expected in a healthy mammary gland with fatty tissue and ductal structures (Figure 2A). In the DMBA group, a high PCNA expression was evidenced by numerous darkly stained nuclei, suggesting increased cell proliferation. The presence of malignant clusters with significant proliferative activity of PCNA expression (Figure 2B) was observed. In the DMBA + skimmianine group (Figure 2C), moderate PCNA expression was observed compared to the DMBA group. There were some positively stained cells but fewer than in the DMBA group, indicating the potential antiproliferative effect of skimmianine, suggesting reduced tumor progression. Statistical quantitative analysis showed PCNA expression was significantly increased in the DMBA group compared to cthe ontrol group (13.0 ± 3.0% vs. 25.0 ± 4.0%, respectively); skimmianine treatment significantly downregulated the expression in the DMBA + skimmianine group (20.0 ± 4.1%) (*p* < 0.0001) (Figure 2D). Our findings indicate that PCNA expression correlates with cell proliferation, and skimmianine may have a suppressive effect on tumor growth.

**Figure 3:**
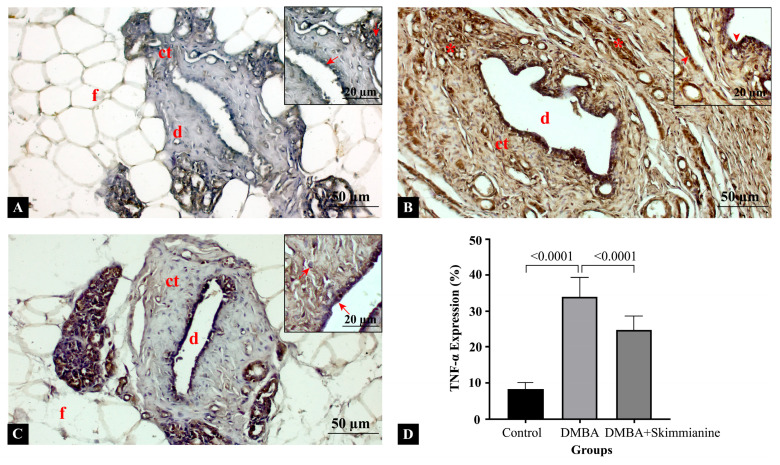
Cross-sections of mammary glands in (**A**) control, (**B**) DMBA, and (**C**) DMBA + skimmianine groups. (**D**) Semi-quantitative measurement of TNF-α expression. Ductal structures (d), connective tissue (ct), malignant cells (*), fatty tissue (f), arrow: negative expression, arrowhead: positive expression. Magnification: 20× (low), 40× (insets).

Mammary gland sections were stained with TNF-α primary antibody to show inflammation (Figure 3). In the control group, there was minimal or no TNF-α expression. Normal mammary gland architecture was observed, with intact ductal and connective tissue. Fatty tissue appeared normal, without a significant inflammatory response (Figure 3A). Compared to the control group, the DMBA group showed strong TNF-α expression, suggesting increased inflammation, with the disruption of ductal and connective tissue structures. Dense cellular infiltration, consistent with a pro-inflammatory and tumor-promoting microenvironment, were evident around ductal structures (Figure 3B). Compared to the DMBA group, skimmianine treatment lowered the TNF-α expression after DMBA induction. Reduced inflammation was observed compared to the DMBA-only group, indicating the potential anti-inflammatory effect of skimmianine. Fatty tissue remained more intact, suggesting a protective effect (Figure 3C). Statistical analysis of the semi-quantitative measurement of TNF-α expression show it was significantly increased in the DMBA group compared to the control group (8.4 ± 1.7% vs. 34.0 ± 5.3%, respectively), while skimmianine treatment significantly downregulated its expression in the DMBA + skimmianine group (25.0 ± 3.9%) levels (*p* < 0.001) (Figure 3D). Skimmianine treatment appears to reduce TNF-α levels, suggesting its potential anti-inflammatory and antitumor effects.

### 2.3. PPI Network and Functional Annotation of Skimmianine-Associated PCNA and TNF-α Targets

**Figure 4:**
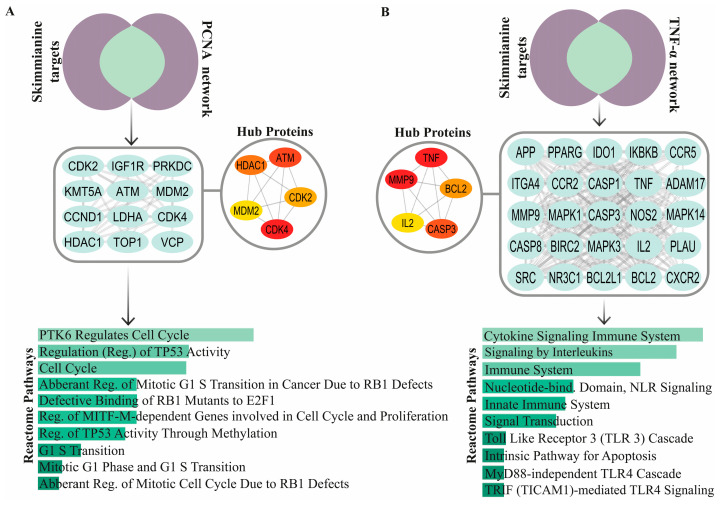
Protein–protein interaction (PPI) network and functional enrichment analysis of skimmianine-associated PCNA (**A**) and TNF-α (**B**) targets. PPI network visualization illustrating hub proteins ranked by importance using the MCC algorithm, where red represents the highest-ranked hub proteins and yellow indicates lower-ranked hub proteins. Functional annotation of shared targets presented as a bar chart, with Reactome pathways ranked by ascending adjusted *p*-values (*p* < 0.05). NLR: Leucine Rich Repeat Containing Receptor.

To investigate the molecular interactions of skimmianine with PCNA, a PPI network was constructed based on common targets. Functional annotation analysis revealed that these shared targets are predominantly associated with cell cycle regulation, DNA replication, and apoptosis. Reactome pathway enrichment analysis identified pathways such as Mitotic G1 Phase and G1/S Transition, Regulation of TP53 Activity, Abnormal Mitotic Cycle Regulation Due to RB1 Defects, and the Intrinsic Apoptotic Pathway (adjusted *p* < 0.05). Hub protein analysis identified key central nodes within the PCNA–skimmianine PPI network, including MDM2 Proto-Oncogene (MDM2), Cyclin-Dependent Kinase 2 (CDK2), CDK4, Ataxia Telangiectasia Mutated (ATM), and Histone Deacetylase 1 (HDAC1) (Figure 4A).

Similarly, a PPI network for TNF-α and skimmianine was constructed to explore their shared molecular targets. Functional annotation analysis indicated that immune signaling, inflammatory response, and cytokine-mediated interactions were the predominant biological processes associated with these targets. Reactome pathway enrichment analysis identified pathways such as Cytokine Signaling, toll-like receptor (TLR) signaling, Nucleotide-binding Domain Leucine-rich Repeat (NLR) Signaling, and TRIF-Mediated TLR4 Signaling (adjusted *p* < 0.05). Hub protein analysis identified TNF-α, CASP3, Matrix Metallopeptidase 9 (MMP9), B-Cell Lymphoma 2 (BCL2), and Interleukin-2 (IL2). Importantly, TNF-α was identified as a central protein in the skimmianine-associated pathways, highlighting its key role in the molecular interactions influenced by skimmianine (Figure 4B).

### 2.4. Immunological Significance of Hub Proteins in the PPI Network of Skimmianine-Shared PCNA and TNF-α Targets

**Figure 5:**
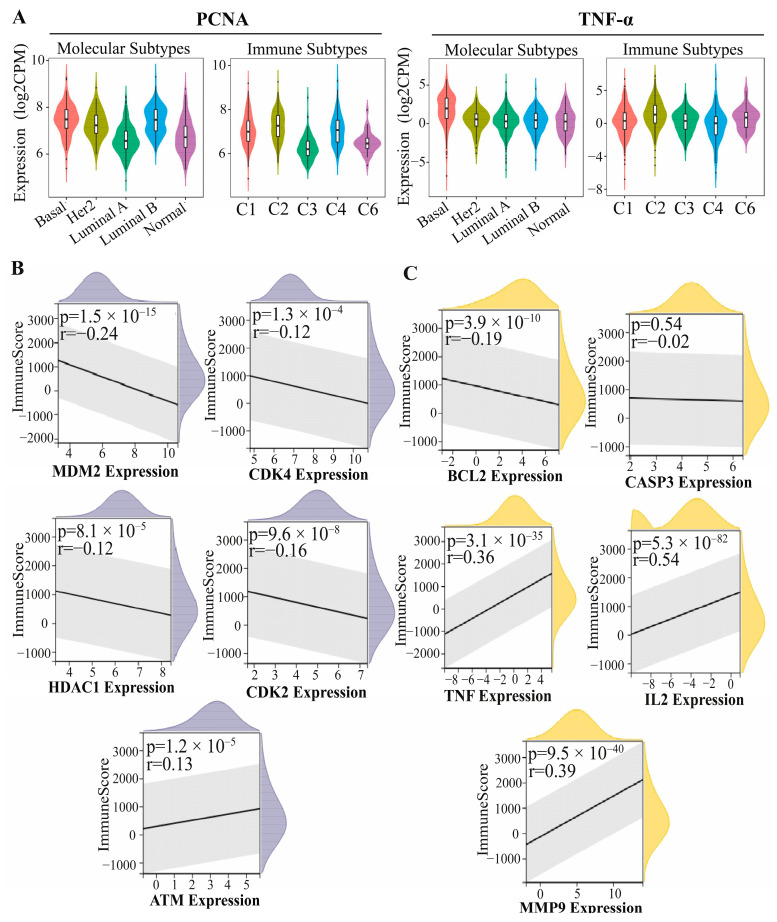
Immunological analysis of PCNA and TNF-α in breast cancer. (**A**) Correlation of PCNA and TNF-α expression with molecular and immune subtypes, showing their increased expression in the basal subtype and their highest expression in the C2 (IFN-γ dominant) immune subtype (CPM: counts per million). ImmuneScore analysis assessing the association between immune modulation and key hub proteins derived from the skimmianine-associated PCNA (**B**) and TNF-α (**C**) PPI networks, identifying significantly correlated proteins (*p* < 0.05).

To comprehensively examine the role of PCNA and TNF-α in breast cancer, molecular subtype correlation analysis was initially performed to determine their relationship with different tumor subtypes. This analysis was conducted to assess whether PCNA and TNF-α are differentially expressed across subtypes. The results showed significant associations, with both PCNA and TNF-α exhibiting a higher expression in the basal subtype of breast cancer (*p* < 0.05). To further investigate the immunological impact, immune subtype analysis was performed to classify tumors into six distinct immune-based clusters (C1–C6). This analysis aimed to determine whether PCNA and TNF-α exhibit distinct expression patterns across immune subtypes, providing insights into their role in shaping immune responses within the TME. The analysis revealed that PCNA and TNF-α exhibited their highest expression levels in the C2 (IFN-γ dominant) subtype, which is characterized by strong immune activation and increased cytotoxic T-cell activity [16] (Figure 5A).

Given these significant immune associations, further analyses were conducted to evaluate the overall immune impact of hub proteins in the PPI network of skimmianine-shared PCNA and TNF-α targets. To achieve this, an ImmuneSCORE analysis was performed to determine whether these proteins are linked to an immune-enriched microenvironment or characterized by high levels of immune cell infiltration or an immune-excluded microenvironment, where immune cells are present but unable to effectively infiltrate the tumor. The results revealed that all skimmianine–PCNA PPI hub proteins exhibited significant immune correlations (*p* < 0.05). Among them, four hub proteins displayed negative correlations (r < 0), while ATM showed a positive correlation (r > 0) (Figure 5B). Similarly, within the skimmianine–TNF-α PPI hub protein network, four hub proteins exhibited significant immune correlations (*p* < 0.05), with BCL2 being the only hub protein showing a negative correlation (r < 0) (Figure 5C). Collectively, the hub proteins within the skimmianine-associated PCNA and TNF-α PPI networks were observed to be linked to the overall immune activity within the TME.

**Figure 6:**
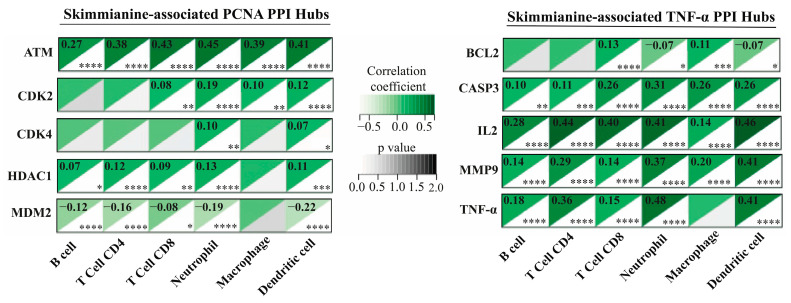
Correlation of skimmianine-associated PCNA and TNF-α PPI hub proteins with tumor immune infiltration. Heatmap analysis depicting the correlation between skimmianine-associated PCNA (ATM, CDK2, CDK4, HDAC1, MDM2) and TNF-α (BCL2, CASP3, IL2, MMP9, TNF-α) hub proteins with six major immune cell infiltrates in breast cancer. In the heatmaps, green regions denote correlation coefficients, whereas black regions represent *p*-values, with significance levels indicated as * *p* < 0.05, ** *p* < 0.01, *** *p* < 0.005, and **** *p* < 0.001.

To further investigate the impact of skimmianine-associated targets on the tumor microenvironment in breast cancer, correlation analyses were conducted between six major immune cell infiltrates and the key hub proteins identified within the skimmianine-associated PCNA and TNF-α PPI networks. All central proteins derived from the skimmianine-associated PCNA and TNF-α PPI networks exhibited statistically significant correlations with at least one of the six major immune cell types (*p* < 0.05). Within the skimmianine-associated PCNA PPI network, all five hub proteins exhibited statistically significant associations with immune infiltration (*p* < 0.05). ATM demonstrated the strongest correlation with all six immune cell types, followed by HDAC1 and MDM2, which also showed substantial associations (*p* < 0.05). CDK4 displayed the weakest correlation among these proteins. Notably, MDM2 was the only hub protein exhibiting a consistently negative correlation with all six immune cell types (*p* < 0.05, r < 0). In the skimmianine-associated TNF-α PPI network, CASP3, IL2, and MMP9 exhibited significant correlations with all six immune cell infiltrates (*p* < 0.05, r > 0). Following these, TNFA exhibited significant associations with five immune cell infiltrates, maintaining a consistent yet slightly lower correlation (*p* < 0.05, r > 0). BCL2 demonstrated the weakest correlation among the TNF-α hub proteins, exhibiting both positive and negative correlations with different immune cell types (*p* < 0.05) (Figure 6).

## 3. Discussion

Breast cancer remains a major global health challenge, necessitating the development of more effective and targeted therapeutic strategies. Skimmianine, a naturally derived furoquinoline alkaloid, has gained attention for its anticancer potential and relatively low toxicity [1,2,3,4,5,17]. In this study, we investigated its effects on breast cancer by evaluating PCNA and TNF-α expression and analyzing their molecular and immunological associations using in silico approaches.

The DMBA-induced breast cancer model is a widely used system that closely mimics human breast tumors, particularly estrogen receptor-positive (ER+) types, due to its high reproducibility and molecular similarity, including frequent Pik3ca and Pten mutations. It is valuable for studying tumor biology, therapeutic agents, and chemoprevention [18]. Moreover, in our study, we evaluated CA15-3 levels, a tumor-associated antigen commonly elevated in breast cancer [19]. to monitor tumor progression, further confirming the model’s reliability in breast cancer research.

Our histopathological analysis demonstrated that skimmianine exerts protective and therapeutic effects against DMBA-induced breast cancer by reducing malignant cell clusters, preserving the mammary gland architecture, and maintaining connective tissue integrity. Unlike the DMBA-induced group, which showed structural disruption and high mitotic activity, skimmianine treatment improved ductal organization and limited tumor invasion. Several studies have demonstrated the anticancer properties of skimmianine across different cancer models. In esophageal carcinoma, skimmianine was shown to inhibit proliferation, migration, and invasion in vitro, while also suppressing tumor growth in vivo, suggesting its potential as an effective antitumor agent [20]. Similarly, in another study, skimmianine exhibited a concentration-dependent antiproliferative effect, suppressing cancer cell proliferation and inducing cell cycle arrest [9]. Additionally, its effects on lung cancer cells revealed significant growth inhibition, indicating a role in apoptosis induction [7]. Moreover, phytochemical investigations on Zanthoxylum chalybeum extracts, which include bioactive compounds such as skimmianine, have demonstrated significant antiproliferative effects against various malignancies, including cervical, gastric, hepatic, and colorectal carcinomas. These anticancer properties have been attributed to the presence of alkaloids such as skimmianine, benzophenanthridine, and furoquinoline [21]. On the other hand, studies investigating the effects of skimmianine in breast cancer remain scarce; however, evidence suggests that it may inhibit cell proliferation, induce apoptosis via caspase-3 activation, and contribute to chemoprevention by modulating the Nrf2 detoxification pathway [9,10]. Consistent with these findings, our biochemical analysis further supports the antitumor potential of skimmianine. In accordance with previous research [22,23], our results demonstrated that DMBA induction led to a significant increase in CA15-3 levels, confirming active tumor progression. Notably, skimmianine treatment effectively reduced CA15-3 levels, highlighting its potential therapeutic role in mitigating the tumor burden. These findings collectively reinforce the therapeutic potential of skimmianine in breast cancer, highlighting its ability to modulate both histopathological and biochemical markers of tumor progression.

Building upon the histopathological findings supporting the anticancer potential of skimmianine, we evaluated its mechanistic effects by examining PCNA and TNF-α expression. These markers were selected due to their established roles in cell proliferation and inflammation, respectively, and their relevance in breast cancer progression. An elevated PCNA expression reflects increased proliferative activity in aggressive tumor phenotypes, while high TNF-α levels contribute to an inflammatory tumor microenvironment that facilitates cancer cell invasion and metastasis [13,14,15,24]. In canine mammary adenocarcinoma, strong PCNA immunoreactivity has been consistently observed, reflecting increased proliferative activity [25,26]. Similarly, in a DMBA-induced breast cancer model in rats, an elevated number of PCNA-positive cells was reported, indicating enhanced cell proliferation [12]. The immunohistochemical staining of mammary gland tumor cells has also shown pronounced PCNA activity, supporting its relevance as a marker of tumor progression [27]. PCNA inhibition has also been explored as a therapeutic target in breast cancer. The small molecule inhibitor AOH1996 targets a cancer-associated isoform of PCNA, suppressing tumor growth without noticeable side effects [28]. Similarly, the PCNA inhibitor ATX-101 has demonstrated anticancer activity in breast cancer models [29]. Our findings displayed significantly increased PCNA expression, consistent with high proliferative activity in malignant clusters; however, skimmianine treatment resulted in moderate PCNA expression, with fewer positively stained nuclei compared to the DMBA group, suggesting its antiproliferative effect.

The suppression of TNF-α may protect against chemically induced breast tumors. A previous study found increased levels of TNF-a in a DMBA-induced breast cancer model [30]. Clinical investigations and in vitro and in vivo animal studies have shown that TNF-α drives breast cancer by inducing the upregulation of oncogenes [14]. In vitro experiments using human mammary stromal cells revealed that TNF-α treatment promotes an inflammatory microenvironment, facilitating tumor progression [31]. Additionally, evidence suggests that TNF-α contributes to the inflammatory setup of breast tumors and enhances tumor activity [32]. Concurrently, inhibitors of TNF-α, such as certolizumab, have been explored in combination with chemotherapy to enhance antitumor efficacy. A Phase I clinical trial combining certolizumab with chemotherapy reported a decreased metastasis and enhanced antitumor activity, emphasizing the role of TNF-α inhibition in reshaping the TME toward cancer suppression [33]. Likewise, skimmianine treatment in our study significantly reduced TNF-α expression, suggesting a potential anti-inflammatory effect, which may partially contribute to the observed histological improvements.

Consistent with our histopathological observations, cell cycle deregulation—especially the transition between G1 and S phases—is frequently reported in breast cancer progression and correlates strongly with morphological disruptions such as increased mitotic activity and nuclear pleomorphism [34,35]. Targeting G1/S regulatory mechanisms not only suppresses tumor proliferation but also restores cellular organization and reduces malignant cell clustering, which aligns with the structural improvements observed in mammary tissues after skimmianine treatment [28]. Furthermore, the activation of TLR signaling pathways in breast cancer has been closely associated with enhanced inflammatory cell infiltration, tissue disruption, and tumor-promoting inflammation within the tumor microenvironment [36,37]. The observed reduction in TNF-α expression of our study, a crucial mediator of TLR signaling, aligns with the reduced inflammatory cell infiltration and improved histopathological architecture seen in the skimmianine-treated group, suggesting that skimmianine may exert protective effects partially by modulating TLR-dependent inflammatory pathways.

To further elucidate the mechanistic effects of skimmianine, pathway enrichment analyses were conducted on its molecular targets, focusing on PCNA- and TNF-α-associated signaling cascades. Pathway enrichment analyses showed that skimmianine primarily targets PCNA-associated signaling pathways, including cell cycle regulation, G1/S transition, mitotic progression, and the p53-mediated DNA damage response. PCNA downregulation by skimmianine may disrupt DNA replication and cell cycle progression, reducing its proliferative potential and limiting tumor growth, consistent with studies showing that targeting PCNA can inhibit DNA replication in breast cancer cells [13]. Additionally, cell cycle inhibition is crucial in breast cancer therapy, as evidenced by the success of CDK4/6 inhibitors like palbociclib, ribociclib, and abemaciclib [34]. TNF-α-associated pathways were also enriched, mainly in immune and inflammatory networks such as cytokine and TLR signaling, which sustain a pro-tumorigenic microenvironment [36]. Skimmianine-mediated TNF-α suppression may contribute to decreased inflammation and immune evasion, aligning with studies showing that TLR4 targeting decreases proliferation and cytokine secretion in breast cancer cells [37]. Moreover, TLR family members have been highlighted as complex regulators in shaping the tumor immune microenvironment in breast cancer, where they can exhibit both pro-tumorigenic and anti-tumorigenic activities [38]. These findings suggest that skimmianine may suppress breast cancer progression by modulating PCNA-related cell cycle control pathways and TNF-α-related TLR signaling pathways.

To comprehensively assess the role of PCNA and TNF-α in breast cancer, their correlation with molecular and immune subtypes was analyzed. Molecular subtype analysis revealed a significant association between both markers and the basal subtype, known for aggressive tumor behavior and a poor prognosis. PCNA overexpression in this subtype aligns with its role in promoting uncontrolled cell proliferation, while elevated TNF-α levels support a pro-inflammatory TME that facilitates disease progression [13,14,15]. Similar findings have been reported previously, highlighting the interaction between proliferative and inflammatory pathways in basal-like breast cancer [35,39]. In the immune subtype analysis, PCNA and TNF-α expression were highest in the C2 (IFN-γ dominant) immune subtype, characterized by heightened immune activation and increased cytotoxic T-cell activity. This suggests that while IFN-γ-driven responses play a crucial role in eliminating tumor cells, the simultaneous overexpression of PCNA and TNF-α may act as an adaptive mechanism for immune evasion and resistance to immune-mediated cytotoxicity. Tumors can develop resistance to TNF-α-induced apoptosis and modulate metabolic pathways, such as enhanced glycolysis, to evade immune responses [40,41]. Additionally, PCNA can interfere with immune regulation by interacting with natural killer (NK) cell inhibitory receptors on cancer cell surfaces, suppressing NK cell-mediated cytotoxicity and promoting immune escape [42]. These findings suggest that skimmianine may interact differently with immune-related mechanisms in basal molecular and C2 immune subtypes of breast cancer, potentially involving PCNA- and TNF-α-associated signaling.

The immune regulatory roles of PCNA and TNF-α in breast cancer were further examined through immune correlation analyses of their skimmianine-associated hub proteins. An ImmuneScore analysis revealed that nearly all hub proteins (except for CASP3) significantly correlated with immune infiltration, emphasizing skimmianine’s broader immunomodulatory potential beyond PCNA and TNF-α. PCNA-related hub proteins (MDM2, CDK2, CDK4, HDAC1) predominantly showed negative correlations with immune infiltration, suggesting their roles in immune suppression, as supported by previous studies [43,44,45]. Their downregulation following skimmianine-mediated PCNA inhibition likely reversed immune suppression, enhancing immune surveillance and cytotoxic activity. Conversely, skimmianine-mediated TNF-α suppression reduced cancer progression, yet TNF-α-associated hub proteins (TNF-α, IL2, MMP9) remained positively correlated with immune infiltration, highlighting the dual role of TNF-α in the TME. While TNF-α enhances immune activation, its overexpression fosters chronic inflammation, driving angiogenesis, EMT, and immune evasion [15]. Thus, a skimmianine-induced reduction in TNF-α expression may be associated with altered inflammatory signaling in the TME, which could play a role in modulating tumor behavior. Overall, skimmianine’s dual targeting of PCNA and TNF-α modulates the immune TME, highlighting its potential as a regulator of immune dynamics in breast cancer by simultaneously reversing immune suppression and mitigating tumor-promoting inflammation.

The immune infiltration analysis was further extended to six immune cell types to deepen the evaluation of skimmianine-associated PCNA and TNF-α hub proteins. Among the PCNA-associated hubs, ATM, HDAC1, and MDM2 showed strong correlations with the infiltration of multiple immune cells, highlighting their roles in tumor–immune interactions. The positive correlation of ATM with immune cell infiltration aligns with its function in DNA damage response and immune activation, as its deficiency is linked to impaired T-cell function and infiltration [46]. Similarly, HDAC1’s positive association suggests a potential role in antitumor immunity, despite its known immunosuppressive functions in breast and other cancers [47,48]. Conversely, MDM2, a negative regulator of p53, was negatively correlated with immune cell infiltration, supporting its role in immune evasion and poor prognosis through the suppression of CD4+ and CD8+ T-cell activity across multiple malignancies, including breast cancer [49]. On the other hand, the majority of TNF-α-associated hub proteins (CASP3, IL2, MMP9, TNF-α) exhibited significant positive correlations with immune infiltration, indicating their involvement in immune activation within the TME. Notably, IL-2 enhances CD8+ T-cell and NK cell function, while TNF-α demonstrates a dual role, promoting both immune activation and chronic inflammation [15,50,51]. Interestingly, although CASP3 did not show strong correlations in a general immune infiltration analysis, it exhibited significant associations specifically with all six major immune cell types, suggesting a more nuanced immune regulatory role, potentially linked to pyroptosis- and/or apoptosis-driven immune modulation [52]. Therefore, our findings collectively indicate that PCNA-associated hub proteins, especially ATM, HDAC1, and MDM2, and TNF-α-associated hub proteins, particularly CASP3, IL2, MMP9, and TNF-α, may act as critical modulators of the infiltration of six major immune cell types within the breast cancer TME, suggesting that skimmianine exerts its immunoregulatory effects by orchestrating their recruitment and activation.

Skimmianine has not yet entered clinical trials for cancer (including breast cancer), and the current evidence is confined to preclinical studies. In vitro, this natural furoquinoline alkaloid exhibits cytotoxic activity against multiple human cancer cell lines by inducing caspase-dependent apoptosis and cell-cycle arrest. It has shown antiproliferative effects in lung, esophageal, and cervical carcinoma models, although its efficacy in breast cancer cells appears modest [7,53]. Mechanistic studies indicate that skimmianine triggers G0/G1-phase cell cycle arrest and inhibits tumor cell migration/invasion, partly via suppression of the MAPK/ERK signaling pathway. Moreover, skimmianine significantly suppressed tumor growth in an esophageal cancer xenograft model in mice [20]. Pharmacologically, skimmianine has a broad bioactivity profile (e.g., anti-inflammatory and acetylcholinesterase-inhibitory effects), supporting its potential utility in drug development. However, like other furoquinoline alkaloids, it can intercalate DNA and has been associated with mutagenic or genotoxic effects [6,54]. Preliminary toxicological studies reported no overt toxicity or organ damage in rodents at moderate doses [17], though its potential genotoxicity remains a concern. Notably, a metabolic profiling study in rats identified numerous skimmianine metabolites and suggested that certain hydroxylated metabolites could contribute to its observed genotoxic effects [55]. For future clinical applications, comprehensive pharmacokinetic and toxicological assessments are needed to fully establish the safety and therapeutic potential of skimmianine in cancer treatment.

Given these pharmacological and toxicological characteristics, evaluating the therapeutic relevance of its molecular targets becomes particularly important. Considering the comprehensive effects of PCNA and TNF-α described throughout this study as primary targets of skimmianine, their suppression underscores their relevance as actionable therapeutic nodes in breast cancer. Given that several preclinical strategies have confirmed the feasibility of targeting PCNA to impair uncontrolled proliferation with tolerable safety profiles, interest continues to grow around disrupting PCNA-dependent replication dynamics in aggressive tumors [29,56]. Importantly, PCNA downregulation may also mitigate immune evasion, as tumor-cell PCNA has been shown to bind the NKp44 receptor on NK cells, thereby suppressing their cytotoxic function [57]. Similarly, the pathological role of TNF-α in driving inflammation-associated tumor progression has made it a rational candidate for combinatory interventions. TNF-α inhibitors, including biologics repurposed from autoimmune indications, have already entered clinical testing in cancer patients, either alone or in combination with chemotherapy or immunotherapy. In breast and lung cancer models, TNF-α has been shown to modulate a chronic inflammatory circuit (TNF-α → CXCL1/2 → MDSC recruitment → S100A8) that disrupts the TME and facilitates chemoresistance and metastasis; interruption at any point in this cascade may markedly reduce tumors’ aggressiveness [33,58]. Moreover, TNF-α neutralization has been reported to prevent TNF-induced T-cell dysfunction and enhance responses to immune checkpoint inhibitors, particularly in preclinical models of melanoma and colorectal cancer [58]. Thus, concurrently targeting PCNA and TNF-α may confer dual antitumor benefits.

Although our study provides the first integrative evaluation of skimmianine’s antiproliferative and immunomodulatory effects in breast cancer, several limitations should be acknowledged. First, while PCNA and TNF-α were selected as representative markers of proliferation and inflammation, skimmianine may also influence other molecular pathways not assessed in this study, including apoptotic, metabolic, or angiogenic signaling cascades. Additionally, the specificity of skimmianine toward different cell types within the TME—such as immune subpopulations or stromal components—remains to be elucidated. Our in vivo model did not include a comprehensive toxicological assessment; therefore, future studies should evaluate the compound’s systemic safety profile, including potential hepatotoxic and nephrotoxic effects, using histopathological and biochemical analyses of liver and kidney tissues. Moreover, mechanistic validation through in vitro knockdown or overexpression studies targeting PCNA and TNF-α could strengthen the causal interpretation of our findings. In addition, the long-term efficacy of skimmianine in sustaining tumor suppression following the cessation of treatment has not been investigated and represents an important direction for future studies. Expanding this work to include human-derived breast cancer models, testing the compound in combination with standard therapies, and exploring its effects in other tumor types such as colorectal and gastric cancers will be essential to determine the broader translational potential of skimmianine. In future studies, addressing these limitations will further strengthen the mechanistic understanding and therapeutic evaluation of skimmianine in breast cancer.

## 4. Materials and Methods

### 4.1. Experimental Design

Ethical approval was obtained from the Local Animal Experimentation Ethics Committee, Dicle University (date: 11 June 2024, issue: 2024/18). In this study, 36 Wistar albino female rats were divided into 3 groups as the control, DMBA group, and DMBA + skimmianine group (*n* = 12 per group). The experimental animals had unlimited access to water and food and were kept under control in a 23 ± 2 °C environment with a 12 h day from 8:00 am to 8:00 pm and 12 h dark. Skimmianine (4,7,8-trimethoxyfuro[2,3-b]quinoline) was commercially obtained from MedChemExpress (catalog no: HY-N2081, purity ≥ 98%, Shanghai, China) and was used without further purification due to its high purity. According to the manufacturer’s instructions, the skimmianine was initially dissolved in a small volume of dimethyl sulfoxide (DMSO, final concentration < 0.1%) and then diluted in a sterile aqueous solution of 5% carboxymethyl cellulose sodium (CMC-Na; catalog no: CAS 9004-32-4, Santa Cruz Biotechnology, Dallas, TX, USA) to achieve the required dose concentration. A fresh stock solution of skimmianine was prepared daily immediately prior to administration to ensure optimal stability and bioavailability. Each rat received intraperitoneal injections of skimmianine at a dose of 40 mg/kg/day in a total volume of 1 mL. The selected administration route (intraperitoneal) was based on previous studies demonstrating the optimal absorption, bioavailability, and consistent systemic distribution of skimmianine and related alkaloids via this route [7,20]. Control animals received an equivalent volume (1 mL) of the vehicle (5% CMC-Na solution containing < 0.1% DMSO) without skimmianine.

### 4.2. Induction of Breast Cancer

Induction of the breast cancer was modified according to the study conducted by Nassan et al. [59]. In our study, a single dose of 50 mg/kg DMBA dissolved in sesame oil was administered orally to the rats. At 16 weeks after DMBA administration, 5% carboxymethyl cellulose sodium aqueous solution was administered orally for 4 weeks.

1. Control Group: Rats in this group were administered 1 mL of sesame oil orally for 16 weeks, followed by 1 mL of 5% carboxymethyl cellulose sodium aqueous solution intraperitoneally for 4 weeks;
2. DMBA Group: Rats in this group were administered 50 mg/kg DMBA-containing sesame oil orally for 16 weeks and 1 mL of 5% carboxymethyl cellulose sodium aqueous solution intraperitoneally for the next 4 weeks;
3. DMBA + Skimmianine Group: After DMBA administration, 40 mg/kg skimmianine dissolved in 1 mL of 5% carboxymethyl cellulose sodium aqueous solution was given intraperitoneally for 4 weeks.

### 4.3. Measurement of Serum CA15-3 Levels

After euthanizing the rats, the blood of the rats was collected for biochemical analysis. Blood samples were centrifuged at 1000× *g* for 10 min at room temperature. The serum was separated and analyzed for tumor markers. Serum CA15-3 levels were measured by commercial ELISA kits (catalog #MBS2502096, MyBioSource, San Diego, CA, USA).

### 4.4. Histological Staining

After the rats were euthanized, their mammary glands were dissected and processed for the routine paraffin wax tissue-embedding protocol. Gland tissue sections obtained from paraffin blocks were deparaffinized in xylene for 3 × 15 min, then rehydrated and stained with hematoxylin–eosin.

An immunohistochemistry with PCNA (sc-25280 Santa Cruz, Dallas, TX, USA) ve TNFα (sc-52746 Santa Cruz USA) was performed according to the biotin–streptavidin peroxidase complex method described previously [60]. Initially, tissue samples were dewaxed in xylene and rehydrated in ethyl alcohol. Endogenous peroxidase activity was blocked by treating the samples with 3% H_2_O_2_ (catalog no: TA-015-HP, ThermoFischer, Waltham, MA, USA) for 20 min. Antigen retrieval was conducted using citrate buffer (pH 6.0) for 10 min at 90 °C. To block nonspecific proteins, a blocking solution (catalog no: TA-015-UB, ThermoFischer, Waltham, MA, USA) was applied for 8 min. Subsequently, the tissue sections were incubated overnight with the diluted primary antibodies, followed by immersion in 1× phosphate-buffered saline (PBS). The sections were then incubated with a biotinylated secondary antibody (catalog no: TP-015-BN, ThermoFischer, Waltham, MA, USA) for 15 min, immersed again in 1× PBS, and incubated with streptavidin peroxidase (catalog no: TS-015-HR, ThermoFischer, Waltham, MA, USA) for another 15 min. The sections were then treated with a chromogen solution (diaminobenzidine, catalog no: TA-001-HCX, ThermoFischer, Waltham, MA, USA) and stained with Gill III hematoxylin for 1 min. The sections were cleaned in xylene and mounted and examined under a Zeiss Imager Axio A2 photomicroscope (Carl Zeiss AG, Wetzlar, Germany).

### 4.5. Semi-Quantitative Histological Scoring

For the semiquantitative assessment of PCNA and TNF-α expression, staining intensity was measured using ImageJ software (version 1.53, http://imagej.nih.gov/ij, accessed on 13 March 2025), according to the protocol described by Crowe et al. [61]. Ten microscopic fields per sample were analyzed in each group, and the quantification results were documented. A brown coloration indicated positive antibody staining, whereas blue represented negative staining. Signal intensity (expression) within each area was obtained by dividing the stained antibody area by the total examined area. For each sample, the ratio of positively stained area relative to the total area was calculated across ten fields, and subsequently, a mean value for each group was determined. These mean values were then used for semiquantitative immunohistochemical scoring.

### 4.6. Measurement of Tumor Burden

The tumor burden was assessed semi-quantitatively based on an histopathological evaluation of mammary gland sections stained with hematoxylin–eosin (H&E) and immunohistochemical markers (PCNA and TNF-α). The presence and extent of malignant cell clusters, architectural disruption, and proliferative index (PCNA expression) were evaluated across 10 representative fields per sample. A rat was considered to exhibit an increased tumor burden if the histological findings demonstrated marked malignant proliferation with an elevated PCNA and/or TNF-α immunoreactivity, as determined by ImageJ-based quantification and histological scoring. The percentage of animals with an increased tumor burden was then calculated for each group based on these criteria.

### 4.7. Integrative Network and Enrichment Analysis of PCNA, TNF-α, and Skimmianine

To investigate the molecular targets and pathways associated with PCNA and TNF-α in relation to skimmianine, protein–protein interaction (PPI) networks for both proteins were retrieved from the Search Tool for the Retrieval of Interacting Genes (STRING) database, using 300 additional interactors and a medium confidence threshold (0.400). Skimmianine-associated protein targets were obtained from ChEMBL, a comprehensive database integrating high-quality, large-scale bioactivity data on bioactive compounds [62]. The PPI networks for PCNA, TNF-α, and skimmianine were then imported into Cytoscape (v.3.10.3.) (Cytoscape Consortium, San Diego, CA, USA), where common protein targets shared between skimmianine, PCNA, and TNF-α were identified and analyzed. A Reactome pathway enrichment analysis was performed using the Enrichr platform to identify biological pathways associated with the common protein targets [63]. The resulting pathways were ranked in ascending order based on adjusted *p*-values, with *p* < 0.05 considered statistically significant. Additionally, key hub proteins within the shared PPI network were identified using CytoHubba in Cytoscape, applying the Maximal Clique Centrality (MCC) algorithm [1,64].

### 4.8. Immune Correlation and Infiltration Analysis

The relationship between PCNA and TNF-α expression and molecular as well as immune subtypes (C1: associated with wound healing, C2: characterized by IFN-γ dominance, C3: linked to inflammation, C4: marked by lymphocyte depletion, and C6: defined by TGF-β dominance) in breast cancer was assessed using TISIDB, a comprehensive tumor–immune system interaction database [65]. To further explore the immunological significance of skimmianine-associated PCNA- and TNF-α-related hub proteins in breast cancer, additional immune-related analyses were conducted to assess their potential roles in tumor immune infiltration and regulation. To quantify the overall immune infiltration linked to skimmianine-related PCNA- and TNF-α hub proteins, an ImmuneSCORE analysis was performed. Subsequently, the Tumor Immune Estimation Resource (TIMER) tool was utilized to evaluate the correlation between hub protein expression and the infiltration of six major immune cell types—neutrophils, macrophages, dendritic cells, B-cells, CD4+ T-cells, and CD8+ T-cells. Expression data for each hub protein specific to breast cancer were extracted from the TCGA, TARGET, and GTEx datasets [66]. All advanced immune-related analyses were conducted using the SangerBox 3.0 platform [67], and correlations with *p*-values < 0.05 were considered statistically significant.

### 4.9. Statistical Analysis

All statistical tests were conducted with IBM SPSS software (version 25) (IBM Corporation, Armonk, NY, USA). The data distribution was tested with the Shapiro–Wilk test. Normally distributed data were tested with an ANOVA (multiple group comparison), followed by a post hoc Tukey test for pairwise comparisons. *p* < 0.05 was accepted as the significance level.

## 5. Conclusions

This study provides initial evidence supporting the potential of skimmianine to modulate tumor proliferation and immune dynamics in breast cancer through PCNA- and TNF-α-associated mechanisms. Histopathological and biochemical analyses revealed its potent anticancer potential, demonstrating the significant downregulation of both markers. Molecular insights from in silico analyses further indicated that PCNA inhibition may affect cell cycle regulatory mechanisms, while TNF-α suppression may influence TLR-associated signaling pathways, suggesting a possible modulatory role of skimmianine in tumor progression. Notably, hub protein analysis identified TNF-α as a direct target of skimmianine, supporting its potential as a key therapeutic target. According to subtype analyses, skimmianine’s PCNA- and TNF-α-associated anticancer effects may be more relevant in basal molecular subtypes and C2 immune subtypes of breast cancer. Additionally, skimmianine may act as a modulator of immune dynamics by contributing to the attenuation of immune suppression and a reduction in tumor-promoting inflammatory processes. Immune infiltration analyses further showed that skimmianine-associated PCNA network hubs (ATM, HDAC1, MDM2) and TNF-α network hubs (CASP3, IL2, MMP9, TNF-α) were associated with the infiltration patterns of six major immune cell types. These findings suggest that skimmianine has the potential to influence the tumor immune microenvironment through the regulation of key molecular hubs involved in immune cell interactions. These results contribute to the current understanding of skimmianine’s potential role in breast cancer and underscore the importance of continued research to clarify its clinical relevance and evaluate its compatibility with existing therapeutic strategies.

## Figures and Tables

**Table 1:** Serum CA15-3 tumor marker per groups.

	Control	DMBA	DMBA + Skimmianine	Multiple Comparisons
CA15-3 (ng/mL)	0.23 ± 0.06	8.57 ± 1.01 *	3.72 ± 0.58 **	<0.0001

Pairwise comparison: * control vs. DMBA: *p* < 0.0001; ** DMBA vs. DMBA + skimmianine: *p* < 0.0001.

## Data Availability

Data are contained within the article.

## Abbreviations

The following abbreviations are used in this manuscript:

PCNA	Proliferating Cell Nuclear Antigen
TNF-α	Tumor Necrosis Factor-alpha
DMBA	7,12-Dimethylbenz[a]anthracene
CA15-3	Cancer Antigen 15-3
H&E	Hematoxylin and Eosin
PPI	Protein–Protein Interaction
TLR	Toll-like Receptor
NLR	Nucleotide-binding Domain Leucine-rich Repeat
TRIF	TIR-domain-containing Adapter-inducing Interferon-β
CDK	Cyclin-Dependent Kinase 2/4
HDAC1	Histone Deacetylase 1
MDM2	Mouse Double Minute 2 homolog
BCL2	B-Cell Lymphoma 2
IL2	Interleukin-2
CASP3	Caspase-3
MMP9	Matrix Metallopeptidase 9
TME	Tumor Microenvironment
NK cells	Natural Killer Cells
Nrf2	Nuclear Factor Erythroid 2–Related Factor 2
STAT3	Signal Transducer and Activator of Transcription 3
HBXIP	Hepatitis B X-Interacting Protein
NF-κB	Nuclear Factor kappa Beta
MCC	Maximal Clique Centrality
TCGA	The Cancer Genome Atlas
GTEx	Genotype Tissue Expression
TISIDB	Tumor and Immune System Interaction Database
TIMER	Tumor Immune Estimation Resource
